# Generation, analysis and functional annotation of expressed sequence tags from the ectoparasitic mite *Psoroptes ovis*

**DOI:** 10.1186/1756-3305-4-145

**Published:** 2011-07-22

**Authors:** Stewart TG Burgess, Alasdair J Nisbet, Fiona Kenyon, John F Huntley

**Affiliations:** 1 Division of Parasitology, Moredun Research Institute, Pentlands Science Park, Bush Loan, Edinburgh, UK

## Abstract

**Background:**

Sheep scab is caused by *Psoroptes ovis *and is arguably the most important ectoparasitic disease affecting sheep in the UK. The disease is highly contagious and causes and considerable pruritis and irritation and is therefore a major welfare concern. Current methods of treatment are unsustainable and in order to elucidate novel methods of disease control a more comprehensive understanding of the parasite is required. To date, no full genomic DNA sequence or large scale transcript datasets are available and prior to this study only 484 *P. ovis *expressed sequence tags (ESTs) were accessible in public databases.

**Results:**

In order to further expand upon the transcriptomic coverage of *P. ovis *thus facilitating novel insights into the mite biology we undertook a larger scale EST approach, incorporating newly generated and previously described *P. ovis *transcript data and representing the largest collection of *P. ovis *ESTs to date. We sequenced 1,574 ESTs and assembled these along with 484 previously generated *P. ovis *ESTs, which resulted in the identification of 1,545 unique *P. ovis *sequences. BLASTX searches identified 961 ESTs with significant hits (E-value < 1E-04) and 584 novel *P. ovis *ESTs. Gene Ontology (GO) analysis allowed the functional annotation of 880 ESTs and included predictions of signal peptide and transmembrane domains; allowing the identification of potential *P. ovis *excreted/secreted factors, and mapping of metabolic pathways.

**Conclusions:**

This dataset currently represents the largest collection of *P. ovis *ESTs, all of which are publicly available in the GenBank EST database (dbEST) (accession numbers FR748230 - FR749648). Functional analysis of this dataset identified important homologues, including house dust mite allergens and tick salivary factors. These findings offer new insights into the underlying biology of *P. ovis*, facilitating further investigations into mite biology and the identification of novel methods of intervention.

## Background

Sheep scab, caused by the mite *Psoroptes ovis *is, arguably, the most important ectoparasitic disease of sheep in the UK in terms of both animal welfare and financial impact. In continental Europe and in the USA, psoroptic mange is also a welfare problem in cattle and is becoming increasingly common in cattle in the UK [1]. Psoroptic mange is highly contagious, causing considerable pruritis and irritation [2]. Current disease control strategies are heavily reliant upon chemotherapy; however concerns over residues, eco-toxicity and the development of parasite resistance threaten the sustainability of current control strategies and have highlighted interest in the development of alternative control methods [3]. Such approaches require a more comprehensive understanding of both the parasite and its interaction with the host.

*P. ovis *is a non-burrowing, surface exudate feeder capable of consuming serous fluids, lymph and red blood cells [4]. Mites survive on the surface of the skin and do not appear to penetrate beyond the stratum corneum, the outermost layer of the skin [5]. The available evidence suggests that mites abrade the stratum corneum and deposit allergens as they progress - the combination of mechanical skin abrasion, mite allergen deposition and increased self-grooming behaviour by the host in response to the pruritis caused by the mites triggers the subsequent activation of a cutaneous inflammatory response [6,7] providing an exudate which supplies the mite with a food source [8]. Establishment of a *P. ovis *infestation is therefore the result of a complex interaction between the host and the mite, during which the mite appears to initiate reactions conducive to its own establishment and maintenance [9]. The skin lesions are induced by mite-derived pro-inflammatory factors, a likely source of which is mite excretory/secretory products, including potent enzymes and allergens (reviewed in [8]). While several sheep scab mite products have been identified, including a number of enzymes and homologues of allergens of the house dust mite (HDM), *Dermatophagoides pteronyssinus *and the scabies mite *Sarcoptes scabiei*, their functions remain largely unknown [10].

Prior infestation with sheep scab mites alters the progression of subsequent infestations and reductions in lesion size have been observed in secondary infestations in sheep, suggesting that vaccination against the parasite may be feasible [11]. Vaccination with *P. ovis *extracts has resulted in a 15-fold reduction in mite numbers and a 4-fold reduction in lesion size [12,13]. However, identification of the individual proteins involved in protective immunity has not yet been achieved. The lack of available *P. ovis *sequence information is hampering vaccine candidate discovery, whether that process is through the pragmatic or rational routes. To date, no full genomic DNA sequence has been available for *P. ovis *and this is the case for most related mite species, i.e. *Dermatophagoides pteronyssinus *and *Sarcoptes scabiei*, where the main focus has been on the generation and analysis of expressed sequence tags (ESTs). To date, only 484 *P. ovis *ESTs have been available in the public databases [14] and, in order to further our understanding of the biology of *P. ovis*, we have now generated a complementary DNA (cDNA) library and have undertaken a larger scale EST approach and incorporated these newly generated and previously-described *P. ovis *ESTs with new bioinformatic analyses to identify further novel mite-expressed genes. This paper describes this approach, which represents the largest such resource for *P. ovis*, along with the functional annotation of the ESTs to further our understanding of this economically important parasite.

## Results and Discussion

### cDNA library and EST analysis

1574 ESTs were derived from a normalised cDNA library constructed from a pool of mixed life stages and mixed sex mites (Library 1). To this dataset we added a further 484 *P. ovis *ESTs previously generated at the Moredun Research Institute (Library 2) [14], giving a total of 2,058 ESTs (Table 1). Filtering for low quality sequences resulted in the removal of 153 ESTs, leaving 1905 sequences for assembly of contigs (93% sequencing efficiency). CAP3 contig assembly resulted in the identification of 255 contiguous sequences (consisting of 2 or more ESTs) and 1290 singletons giving a total of 1545 unique sequences (Table 1). Overall sequence length including both contigs and singletons ranged from 187 bp - 1553 bp, with a mean length of 700 bp (contigs: mean length = 800 bp, range = 397 bp - 1553 bp; singletons: mean length = 675 bp, range = 187 bp - 922 bp). All new ESTs generated here were submitted to the EST database (dbEST) at GenBank under the accession numbers FR748230 - FR749648. BLAST analysis of the 1545 assembled ESTs identified 961 (62%) with significant BLAST hits. Of these, 192 represented contiguous sequences, whilst the remaining 769 were singletons; summary details of the largest contigs (Top 10) are described in Table 2. E-values for the 961 ESTs ranged from 1.0E-4 to 3.6E-180. The mean percentage similarity score between and EST and its closest homologue was 68.4%, ranging from 36% - 100%. The most abundant phylum associated with the top BLAST hits was that of the arthropoda, including insects, i.e. *Drosophila melanogaster*, a number of ticks, i.e. *Ixodes scapularis *(deer tick) and other mites, i.e. *Blomia tropicalis, D. pteronyssinus *and *D. farinae*. The top BLAST hit species was *I. scapularis *which represents the most closely related organism to *P. ovis *for which a fully sequenced genome exists. The remaining 584 ESTs with either no BLAST hit or non-significant hits may represent novel *P. ovis *transcripts, of as yet undefined function.

**Table 1:** *P. ovis *EST analysis and breakdown of processing stages

Sequence Description	Number of ESTs
cDNA Library 1	1574
cDNA Library 2	484
**Total ESTs**	**2058**
Removal of low quality sequences	-153
**Total remaining ESTs**	**1905**
CAP3 Contigs	255
Singletons	1290
**Total ESTs**	**1545**
Significant BLAST hits	961
No or non-significant BLAST hit	584

Summary of *P. ovis *EST processing steps and results of BLAST search.

**Table 2:** Details of the largest contigs from the *P. ovis *EST dataset

Contig ID	Contig Size (bp)	No. of ESTs represented	Example EST ID from Contig	Top Blast hit
Contig 63	1553	2	Bu_007_c09	Karyopherin alpha 6
Contig 95	1529	4	Bu_012_f01	GTP-binding protein SAR1B
Contig 28	1520	4	Bu_004_g08	T-complex protein 1 subunit gamma
Contig 195	1481	4	Bu_003_a06	Mitochondrial ribosomal protein I43
Contig 78	1435	5	Bu_010_d06	Chitinase
Contig 246	1322	3	Bu_010_a01	WD repeat and FYVE domain containing 2
Contig 107	1310	2	Bu_013_c11	RAS-related GTP binding A
Contig 236	1297	3	Bu_011_d04	Hypothetical protein ISCW014890 (*Ixodes scapularis*)
Contig 71	1226	3	Bu_009_g11	NIPSNAP1 protein
Contig 121	1201	2	Bu_014_f12	Sphingomyelinase D-like protein

Summary details of the top 10 largest contigs from the *P. ovis *EST dataset. Example EST ID refers to the individual EST ID from the *P. ovis *EST dataset.

### Functional annotation of *P. ovis *ESTs

#### Gene Ontology (GO) term annotation and mapping

**Figure 1:**
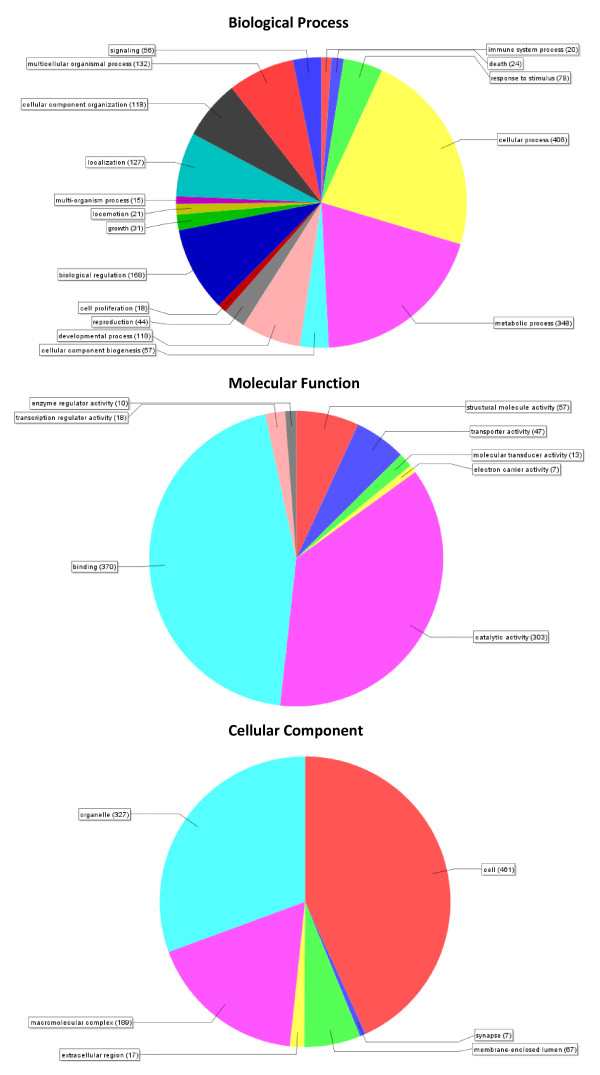
**Pie charts demonstrating distribution of second-level GO terms across the three major GO categories**. Top pie-chart represents the GO category Biological Process, middle chart represents Molecular Function and the bottom chart represents Cellular Component. Number in brackets represents the number of *P. ovis *ESTs associated with each GO category. NB: Each individual EST may be associated with more than one GO category.

In order to gain a functional understanding of the assembled ESTs Gene Ontology (GO) annotation was associated with each of the sequences within the blast2go application [15-17]. This process involved mapping the BLAST hits against known GO terms and then further annotation of the selected GO terms by integrating data from a range of sources including; InterPro to derive protein families and domains; Enzyme Commission (EC) codes to determine distinct functional enzyme class data and Kyoto Encylopedia of Genes and Genomes (KEGG) to identify enriched signalling pathways. Of the 961 ESTs with significant BLAST hits, 880 were associated with GO terms. The majority of sequences were associated with more than one GO term (n = 743), with some being associated with as many as 50 individual GO terms. Only 81 ESTs (8.4%) could not be mapped to a particular GO annotation. The GO terms were split over the 3 main categories [Biological Process (BP), Molecular Function (MF) and Cellular Component (CC)] with the majority in the BP and MF categories. In order to visualise the GO terms represented by the ESTs we mapped the second level GO terms across all three categories (BP, MF and CC) and these are presented as percentage pie-charts in Figure 1. In the BP category the majority of second level GO terms were associated with either cellular (23%) or metabolic processes (20%), in the MF category the majority of GO terms were associated with either catalytic activity (37%) or binding processes (45%) and in the CC category most were associated either with the cell (43%) or organelle (31%). Also of note in the BP category were terms associated with locomotion, reproduction, growth, biological regulation, developmental process, immune system process and response to stimulus. In the MF category a number of sequences were found to be associated with enzyme and transcriptional regulator activity and molecular transducer activity. A multi-level GO term analysis provided further insights into EST function, revealing the nature of the binding and catalytic activities highlighted at the second level of GO terms in the MF category. A number of enzyme classes were identified including protein kinases, ligases, oxidoreductases, hydrolases and endopeptidases. Amongst the binding activities identified were ATP, GTP, RNA and DNA binding, as well as zinc ion binding, co-factor binding and cytoskeletal protein binding. Multi-level GO term analysis of the BP category revealed a number of interesting associations, of particular note were reproductive developmental processes, signalling pathways, cell development, cytoskeleton organisation, nervous system development, proteolysis and response to stress and chemical stimulus. Multi-level analysis of the CC category revealed a number of sequences with terms associated with the cytosol, nucleoplasm, cytoskeleton, plasma membrane, lipid particles and integral membrane molecules.

#### Identification of excreted/secreted proteins

Interpro consists of a collection of protein family and domain signatures and can be used for the automatic annotation of proteins [18]. The Interproscan software tool allows the scanning of protein sequences against the Interpro database using a range of profile-based hidden Markov models (pHMM) and positional specific score matrix protocols and in this way overcomes the problems inherent in single database searches [19]. Of the 961 ESTs with significant BLAST hits, Interpro was able to map 849, leaving 112 with no further protein domain mapping. 533 ESTs were found with both GO term mapping and Interpro mapping, providing both gene onotology and protein domain information, whilst 316 ESTs were successfully mapped by Interpro but not to a specific GO. Interproscan incorporates searches for structural and functional annotation of protein sequences, along with prediction of signal peptide sequences (SignalP) and transmembrane helices (TMHMM) [18,20,21]. Potential secretory proteins, secreted through the classical pathway, can be identified through the presence of a signal peptide sequence [22]. This sequence targets proteins for translocation across the ER membrane and is an N-terminal peptide, typically 15-30 amino acids long which is cleaved during translocation [22]. Not all proteins with SPs are secreted and some may become integrated into the plasma membrane, i.e. transmembrane [22]. This type of profiling can help to identify potentially secreted/excreted peptides and can aid in the identification of signalling pathways and potential diagnostic or therapeutic targets [20]. Interproscan revealed the presence of 173 ESTs with predicted signal peptides (SP) and 191 sequences with a predicted transmembrane domain (TMD). By filtering the SP sequences against the TMD sequences we were able to identify 74 ESTs with a predicted SP but no TMD, thus representing potential *P. ovis *excretory/secretory proteins. A further 183 ESTs were identified with predicted SP and TMDs along with a predicted glycophosphatidylinositol (GPI) anchor sequence thus representing potential *P. ovis *excreted/secreted outer membrane proteins. A final group of 129 ESTs was identified with no predicted SP but with predicted TMD and GPI anchor sequence and this group may represent *P. ovis *outer membrane proteins. The identification of a number of *P. ovis *sequences predicted to encode either secreted or membrane integrated proteins is an important step in the elucidation of mite factors as potential therapeutic targets. By their nature, secreted and/or membrane bound factors are more likely than cytosolic factors to be encountered by the host immune system and may therefore represent valid therapeutic or diagnostic targets. As *P. ovis *is a non-burrowing mite it is thought to rely on the action of a number of proteases to trigger a host inflammatory response to produce an inflammatory exudate, upon which it is then able to feed [8]. It is highly likely that some of these proteases are either secreted or excreted, for example the HDM protein Der p 2 (of which Pso o 2 is a homologue) has been shown to be derived from cells which line the mite gut and has been found in mite faecal pellets [23]. Further interrogation of these ESTs may lead to the identification of novel *P. ovis *E/S factors and therefore new therapeutic or diagnostic targets.

#### Enzyme code annotation

Of the 961 ESTs with significant BLAST hits, 223 (23%) mapped to a specific Enzyme Commission (EC) code. Of these, 126 mapped to unique EC codes and a number of enzyme classes were represented, including oxidoreductases (n = 52), transferases (n = 50), hydrolases (n = 99), lysases (n = 4), isomerases (n = 5) and ligases (n = 13) (Table 3). Thirty nine ESTs mapped to a single EC code, 3.6.5.3, which represents protein-synthesising GTPases. These enzymes are involved in mRNA translation into protein by the ribosome and include translation initiation factors. This process is involved in the regulation of gene expression by controlling translation rates and therefore could be involved in a number of processes including the control of stage-specific gene expression in *P. ovis *[24]. A number of *P. ovis *enzyme activities have previously been identified; most of these are proteolytic and involve cysteine, metalloproteinases and aspartyl proteases [25]. These enzymes are able to digest a number of key protein substrates, including fibronectin, haemoglobin, and fibrinogen and may be involved in mite feeding [25]. Amongst the ESTs analysed here we have confirmed the presence of these enzyme classes and also identified transcripts encoding a number of additional proteases, namely dipeptidase (n = 1), aminopeptidase (n = 1), serine-type carboxypeptidases (n = 3), metallocarboxypeptidase (n = 1), metalloendopeptidases (n = 2), threonine endopeptidases (n = 6) and two further proteases (nedd88 and a prolyl endopeptidase). The cathepsins identified here represent *P. ovis *cysteine proteases and may have roles in mite digestive processes as has been shown for a cathepsin L-like molecule potentially involved in meal digestion in the cattle tick *Rhipicephalus microplus *[26]. Digestive system-derived proteases are likely to be important in food processing in the mite and, as with the aspartyl proteinase Na-APR-1 of hookworms, may therefore represent highly effective vaccine candidates. In addition, mite derived cysteine proteinase molecules may also interact with host keratinocyte-derived cystatin-A, which is a cysteine protease inhibitor produced as part of the host cutaneous immune response to mite-derived allergens [27].

**Table 3:** Summary of EST dataset

Gene name	Putative function/enzyme class	Homologous protein in house dust mite	Top species hit	BLASTX E-value	EST length (bp)	Similarity (%)
Enzymes						
Cathepsin B	Cysteine protease	-	Ixodes scapularis	5.9E-83	1110	67
Cathepsin L	Cysteine protease	-	Suidasia medanensis	6.9E-19	541	53
Thioredoxin-1	Thioredoxin	-	Eriocheir sinensis	1.5E-21	751	76
Glutathione S transferase delta class 2	Glutathione-S-transferase	-	Dermatophagoides pteronyssinus	6.5E-45	569	67
Peroxiredoxin-2	Peroxiredoxin	-	I. scapularis	1.4E-25	771	85
FK506-binding protein (cyclophilin-like)	Cyclophilin	-	Drosophila persimilis	5.1E-48	646	90
Allergens						
Pso o 1	Cysteine protease	Der f 1/Der p 1	D. farinae	9.36E-90	735	87
Pso o 2	MD-2-like	Der f 2/Der p 2	D. farinae	2.4E-77	625	87
Pso o 3	Serine protease	Der f 3/Der p 3	Euroglyphus maynei	1.4E-62	801	73
Pso o 5	Unknown	Der f 5/Der p 5	D. farinae	5.3E-35	598	80
Pso o 7	Unknown	Der f 7/Der p 7	D. farinae	9.6E-41	710	70
Pso o 13	Unknown	Der p 13	D. pteronyssinus	1.1E-13	712	60
Pso o 21	Unknown	Der f 21/Der p 21	D. farinae	2.4E-36	624	78
Tick homologues						
Fed tick salivary protein 5	Small heat shock protein	-	I. scapularis	5.2E-14	763	59
Secreted salivary gland protein	Secreted protein	-	I. scapularis	1.3E-7	679	47
Heat shock protein 20.6	Heat shock protein	-	I. scapularis	4.7E-64	1103	75
Group 10 secretory phospholipase A2	Phospholipase	-	Nasonia vitripennis	1.6E-31	748	59
Chitinase	Chitinase	-	Saccharopolyspora erythraea	5.7E-71	1435	54
Peritrophic membrane chitin binding protein	Chitin binding protein	-	Culex quinquefasciatus	7.4E-59	1073	51
Others ESTs of interest						
Macrophage migration inhibition factor	Lymphokine	-	Amblyomma americanum	6.5E-21	945	60
Spermatogenesis associated factor	Unknown	-	Anopheles gambiae	1.1E-27	604	72
Cytochrome P450	Oxidation	-	Saccoglossus kowalevskii	1.7E-25	659	60

Table shows sequences encoding for proteins with homology to house dust mite allergens, tick proteins and enzymes of interest to *P. ovis *biology.

Additional EC classes identified included thioredoxins, glutathione S transferases (GSTs), peroxiredoxins and cyclophilins (immunophilins). Thioredoxins and peroxiredoxins are antioxidant enzymes which could form a part of the mites defence against reactive oxygen species (ROS) generated during the host inflammatory response. Homologous enzymes have been identified in HDMs and have been implicated in the scavenging of ROS and depression of superoxide dismutase, glutathione peroxidise and catalase activities [28,29].

A number of GSTs were identified from the *P. ovis *ESTs described here and these include delta, mu and alpha classes. GSTs catalyse a variety of enzymatic reactions but perhaps the most relevant is the detoxification and breakdown of xenobiotic substances and these enzymes may be involved in the mites' defence against a range of environmental and host derived compounds. For example, increased transcription of mu, and delta class GSTs has been demonstrated in *S. scabiei *mites following exposure to the acaricidal compound permethrin [30]. GSTs have also been shown to react strongly with host IgE and IgG suggesting a further role in the development of the allergic immune response [31-33].

Two ESTs encoding scavenger receptor cysteine rich protein (SRCRP) and cysteine rich venom protein with trypsin inhibitor like (TIL) domains [34] were also identified. Secretion of proteins with trypsin inhibitory domains may increase mite survival by blocking host immune molecules or by interfering with proteolytic actions, as has been previously described for a TIL domain-containing peptide from the cattle tick *Rhipicephalus microplus *[35]. This factor may also regulate the actions of proteases secreted by the mites themselves. The physiology of the skin and the integrity of an effective skin barrier are maintained by the correct balance of protease and anti-protease activities [36] -the disruption of barrier function through the interruption of the fine balance in protease activity may represent one means by which mites achieve allergen infiltration and thus immune activation. The identification here of a number of additional enzyme classes from *P. ovis *has further expanded our current understanding of the enzymatic repertoire of this economically important ectoparasite and this knowledge will aid in the identification of novel means of targeting *P. ovis *and interfering with the host-parasite relationship.

#### Allergen homologues

A number of *P. ovis *antigens/allergens have previously been identified through their homology with molecules from other mite species, for example the HDM, *D. pteronyssinus *and the scabies mite *S. scabiei *[37,38]. Among these are homologues of the HDM allergens Der p 1 (termed Pso o 1), Der p 2 (Pso o 2), Der p 5 (Pso-der p V-1), Der p 7 (Pso-gp7-1), Der p 10 (tropomyosin), Der p 11 (paramyosin) and Der p 14 (M177/apolipophorin/vitellogenin) [25,39]. Whilst confirming the presence of these homologous genes in *P. ovis*, the EST analysis described here has also led to the identification of additional HDM homologues, namely Der p 21 (Pso o 21), Der p 3 (Pso o 3) and Der p 13 (Pso o 13) which may represent *P. ovis *allergens (Table 3). The action of a number of these allergens has been widely described in the literature for HDMs (for a review see [10]), however, homologues of Der p 3 and Der p 21 have not been previously described in *P. ovis*. Der p 21 is a 15 kDa allergenic protein which is excreted in HDM faecal pellets and has been associated with allergic asthma [40]. HDM Der p 3 allergen is a 30 kDa serine protease with trypsin-like activity that is able to enzymatically digest the complement components C3 and C5, generating the anaphylatoxins C3a and C5a, thus contributing to the pathogenesis of allergic disease [41]. We have previously demonstrated the involvement of the complement system in the host response to *P. ovis *[6] and the identification of a Der p 3 homologue in *P. ovis *adds further support to this finding. The identification of a Der p 21 homologue is a significant finding and could indicate a novel mechanism by which *P. ovis *interacts with the host immune response. The confirmation of the presence of these highly conserved allergens across a number of mite species indicates that they are likely to perform functions that are crucial to mite biology and survival, which marks them out as prime targets for intervention.

#### Tick homologues

The species with the highest representative number of top BLAST hits was the deer tick *Ixodes scapularis*. In recent years a large number of ESTs have been recorded for this parasitic tick in public databases, and it probably represents the most studied, closely related species to the *P*. ovis. We identified a number of homologues of tick proteins including four homologues of tick secreted salivary gland proteins, ten tick heat shock proteins including a homologue of the fed tick salivary protein 5 (FTSP5), which has been shown to be upregulated in a number of invertebrate pathologies and may be involved in the response to hypoxia and oxidative stress [42,43], and a Group 10 secretory phospholipase A2 (PLA2G10) homologue (Table 3). Of particular interest is the presence of homologues of fed-tick and secreted salivary gland proteins, as the secretion of proteins and potential allergens by *P. ovis *has been postulated, but not proven to date, neither has the existence of a salivary gland or similar structure. The identification of *P. ovis *salivary proteins representing possible allergens is intriguing from a control perspective as their secretory nature makes it highly likely that these molecules would be seen by the host immune response. Further investigation of these factors may identify secretory/excretory pathways in *P. ovis *that could be exploited as diagnostic or therapeutic targets. Additional ESTs which may have a bearing on key aspects of mite biology included a homologue of a tick chitinase and other chitin molecules, one of which shared homology with a peritrophic membrane (PM) chitin binding protein. The PM is composed of chitins and proteins and forms a film-like structure which separates food from the midgut tissue in a number of arthropod species [44]. Therefore this PM molecule could potentially play a role in the formation of the mite gut digestive architecture. We also identified 4 homologues of tick cuticle proteins and it is likely that these are involved in the development of the mite exoskeleton.

#### Additional ESTs of interest

A number of other mite proteins that could be involved in the host-parasite interaction in sheep scab were also identified, including a homologue of macrophage migration inhibition factor (MIF) from the lone star tick *Amblyomma americanum*. In humans, MIF encodes a lymphokine involved in cell-mediated immunity and the regulation of inflammatory responses [45]. Homologues of MIF have been identified in a number of parasites including the malaria parasite *Plasmodium falciparum *[46] and are thought to play a role in manipulation of the host immune response to enhance parasite survival in a number of species of helminth parasites. A homologue of a schistosome major egg antigen was also identified and this may prove to be of interest as egg development factors represent potential therapeutic targets [47]. Other factors found included four ESTs involved in spermatogenesis and sperm motility, these factors could form future targets for chemical/therapeutic control of sheep scab as disease pathogenesis relies upon successive rounds of mite reproduction [7]. Of interest was the discovery of 23 homologues of cytochrome subunit proteins (2.4% of the total ESTs), including a number of cytochrome P450 (CYP) factors which are one of the major factors involved in drug metabolism [48]. These may be involved in the processing of substances and compounds that are toxic to the mites, i.e. acaricidal compounds, and may therefore be important from the perspective of parasite drug resistance. The increasing dependency on macrocyclic lactone compounds to treat sheep scab combined with their continued use in the treatment of gastrointestinal nematode infections means that the future development of resistance to these compounds is inevitable. Resistance to the insecticidal compounds flumethrin and propetamphos has already been reported in *P. ovis *and resistance to ivermectin has also been recently reported [49-51]. A better understanding of the mechanisms involved in the processing of toxic compounds by *P. ovis *will allow the design of novel therapeutic targets and may also enable the identification of genetic markers of resistance in the mite population.

#### KEGG pathways

**Figure 2:**
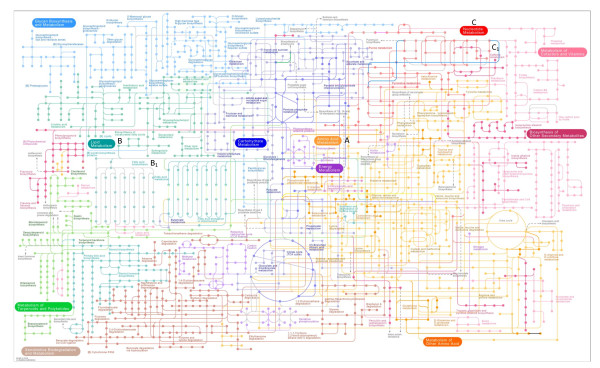
**Graphical representation of the *P. ovis *EST metabolome derived from amalgamated KEGG pathways**. Mapping of peptides predicted from the *P. ovis *EST dataset to known metabolic pathways from the KEGG database. Nodes (circles) represent compounds and edges (lines) represent enzymatic reactions. Distinct metabolic pathways are denoted by a colour-coding scheme, i.e. Amino acid metabolism (light orange and marked A on the pathway map) and Lipid Metabolism (Teal and marked B). Sub-pathways follow the same colour-coding scheme, i.e. Fatty acid biosynthesis (Teal and marked B_1_). Specific enzymes identified within the *P. ovis *EST dataset can be observed where an edge is coloured differently from that of the background metabolic pathway, i.e. Enzyme: 2.7.7.6 which is a DNA-directed RNA polymerase and catalyses the 3' extension of RNA (represented by the blue line marked C_1 _within the Nucleotide metabolism pathway (coloured red by default and marked C on the pathway map). For ease of interpretation a zoomable reference pathway map is available using the free software package Pathway Projector at the following website which allows further investigation of individual enzyme reactions: http://ws.g-language.org/g4/.

To identify signalling and metabolic pathways enriched within the *P. ovis *EST set, annotated sequences were mapped against the KEGG pathway database [52]. This analysis identified 78 enriched pathways within the current *P. ovis *transcriptome, ranging from pathways with a single member represented by a *P. ovis *EST, i.e. N-Glycan biosynthesis, to the purine metabolism pathway represented by 21 individual *P. ovis *ESTs (Table 4). Figure 2 shows a representation of the mapping of the peptides inferred from the *P. ovis *transcriptome to known metabolic pathways and highlights the complex biology of this parasite. From within this map individual pathways of interest can be identified and include metabolic pathways for amino acids, lipids, energy, nucleotides and carbohydrates and also a number of pathways involved in xenobiotic biodegradation and metabolism. Of particular interest are pathways involved in amino acid metabolism, which represents 11 individual pathways; oxidative phosphorylation (the pathway in which the most individual members were identified), fatty acid and drug metabolism. Amino acids are critical components of mite biology and it is clear from the pathways highlighted here that amino acid metabolism in *P. ovis *involves a multitude of enzymes spread over many separate and interconnected pathways. The most enriched pathway identified here was that of oxidative phosphorylation. This is a metabolic pathway involved in the oxidation of nutrients to produce ATP as an energy resource [53]. We identified a number of predicted *P. ovis *enzymes involved in this pathway indicating its importance to the fundamental biology of the mite. The synthesis and metabolism of fatty acids is a crucial stage in the growth and development of all parasites and the fatty acid metabolism pathway was enriched in the *P. ovis *transcriptome, with 6 potential *P. ovis *enzymes identified as being involved in this process.

**Table 4:** Top 10 enriched KEGG pathways and number of ESTs represented

KEGG Pathway	No. of *P. ovis *ESTs represented
Oxidative phosphorylation	24
Purine metabolism	21
Biosynthesis of alkaloids	21
Biosynthesis of plant hormones	15
Methane metabolism	10
Biosynthesis of phenylpropanoids	9
Valine, leucine and isoleucine degradation	8
Biosynthesis of terpenoids and steroids	7
Glcerophospholipid metabolism	7
Fatty acid metabolism	6

Summary of the top 10 KEGG pathways enriched for in the *P. ovis *EST dataset. Also listed is the number of *P. ovis *ESTs represented in each pathway.

Two predicted *P. ovis *enzymes identified here were a flavin-containing monooxygenase (FMO) and a uridine 5'-diphospho-glucuronosyltransferase (UDP-glucuronosyltransferase, UGT). Both enzymes are involved in the catalysis and processing of xenobiotics and are crucial for the elimination of toxic foreign chemicals and drugs from the body [54,55]. Pathway enrichment analysis of *P. ovis *ESTs has two distinct advantages, the first is to enable the identification of the signalling pathways involved in the basic mite biology and the second is to give greater confidence in these findings as they are based upon functional groupings of ESTs and not on single ESTs in isolation. We have described the identification of members of a number of key biological pathways in the mite and we have also described these pathways in the context of a much greater pathway, the *P. ovis *metabolome.

## Conclusions

Here we have described the generation and analysis of 1545 unique and assembled EST sequences for the ectoparasitic mite *P. ovis *from a mixed pool of all life and sex stages. This is by far the largest such resource available for this parasite so far. The majority of these sequences (961 ESTs, 62%) showed similarity to known genes allowing an in depth functional analysis of the *P. ovis *transcriptome. 584 ESTs (38%) showed no similarity to known sequences, these may represent novel, as yet uncharacterised *P. ovis *transcripts and this finding highlights the current dearth of mite EST sequences available in the public databases. During this study we identified a number of important homologues, including HDM allergens and tick salivary factors which offer new insights into the underlying biology of the mite and also into the host-parasite relationship. It is anticipated that further exploration of the host:parasite relationship in sheep scab will enable the development of new methods of parasite control, i.e. identification of homologous mite allergens suitable for use as vaccine candidates and may also aid development of novel drug targets.

The generation of this EST resource will also enable the production of *P. ovis *cDNA microarrays for further analysis of gene expression in these mites and other closely related species. This resource will also allow the transcriptional analysis of different life cycles stages, i.e. egg, larva, nymphal stages and adults and analysis of the consequences of drug exposure, thus facilitating investigations into mite virulence and drug resistance mechanisms.

## Methods

### *P. ovis *mite collection

Ethical approval for this study was obtained from the Moredun Research Institute Experiments Committee and animals were monitored daily in accordance with guidelines agreed with the UK Home Office. *P. ovis *mites (a mixed population consisting of adults, nymphs and larvae) were harvested from infected donor animals maintained at the Moredun Research Institute as previously described [6] and care taken to remove contaminating skin debris by extensive washing. For the construction of the *P. ovis *cDNA library 800 mg of mites were immediately snap frozen in liquid nitrogen and stored at -80°C for later RNA extraction.

### Extraction of total RNA from *P. ovis*

Total RNA was isolated from mites as described previously [41]. RNA samples were further purified using a Qiagen RNeasy kit, following the manufacturer's RNA cleanup protocol and on-column DNase I digestion for 15 minutes at room temperature, before elution into RNase free dH_2_O. *P. ovis *RNA yield was assessed on a ND-1000 Nanodrop spectrophotometer (Thermo Scientific, UK) and RNA sample quality assessed on an Agilent Bioanalyser (Agilent, UK) and RNA Integrity Number (RIN) obtained for each sample. The RIN value is based on a ratio score of the 18s and 28s ribosomal RNA (rRNA) peaks, unfortunately RINs could not be obtained from the *P. ovis *RNA because, as is the case for many arthropods, the 28s rRNA species in *P. ovis *migrates with the 18s rRNA [56]. However the presence of a clean prominent peak for the 18s rRNA and no obvious degradation product indicated that the *P. ovis *RNA was of both high quality and purity.

### Construction of a *P. ovis *mite cDNA library

A normalised *P. ovis *cDNA library was constructed from total RNA extracted as detailed above (Eurofins MWG, Germany). The cloning vector used was pBluescript II sk+ and the cloning sites were 5' EcoRI and 3' BamHI, incorporating 5' and 3' adapter sequences. The bacterial strain used was NEB 10-beta. The library titre was estimated to be 1150 cfu/μl, the total number of clones was estimated to be 2.3 million and the average insert size was ~0.9 kb. Normalisation was carried out by denaturation and subsequent reassociation of the cDNA strands.

### Sequencing of clones

The cDNA inserts of randomly selected clones were amplified by polymerase chain reaction (PCR) using M13 primers and sequence was obtained for 1574 randomly selected clones (Eurofins MWG, Germany). A further 484 *P. ovis *ESTs generated in a previous study by our group (accession numbers in the NCBI dbEST database from BQ834597.1 - BQ835080.1) were then added to the 1574 ESTs described above resulting in a total of 2,058 sequences for downstream analysis [14].

### Bioinformatical analysis

Sequences were checked and filtered to remove low quality sequence data (EST length < 100 bp), vector and adaptor sequences and to mask polyA sequence using the vector tools at EMBL-EBI (http://www.ebi.ac.uk/Tools/sss/ncbiblast/vectors.html). ESTs with an overlap of > 50 bp were assembled into contiguous sequences "contigs" using the CAP3 sequence assembly program [57]. In order to identify homologs of the assembled *P. ovis *EST sequences, all singletons and contigs were BLAST searched against the Genbank non-redundant nucleotide and amino acid databases using the BLASTX and BLASTN algorithms within the blast2go application [15-17,58]. Sequences with non-significant or no BLAST score [Expectation (E) value ≤1.0E-4 and/or no BLAST hit] were excluded from further analysis. E-value describes the number of hits expected by chance when searching a database of a particular size, the lower the E-value the more significant the match [58].

### Functional classification of *P. ovis *ESTs

Putative biological functions and gene ontology classifications (GO) were assigned to the unique ESTs using the mapping and annotation functions in the blast2go package [15-17]. In addition ESTs were mapped to metabolic pathways and where available Enzyme Commission (EC) codes and Interproscan IDs assigned using the respective options in blast2go [15-17]. Potential signal peptide domains were identified using the SignalP 3.0 server, using both the Neural Networks and Hidden Markov Models algorithm [59] and predicted transmembrane domains were identified using the TMHMM 2.0 server (http://www.cbs.dtu.dk/services/TMHMM/) both from within the blast2go package.

## Competing interests

The authors declare that they have no competing interests.

## Authors' contributions

STGB designed the study, performed and processed samples and data analysis and wrote the manuscript. AJN participated in the study design and data analysis and helped prepare the manuscript. FK generated a *P. ovis *cDNA library, helped with data interpretation and preparation of the manuscript. JFH conceived and designed the study, participated in the analysis and helped prepare the manuscript. All authors have read and approved the manuscript
